# Incremental role of glycaemic variability over HbA1c in identifying type 2 diabetic patients with high platelet reactivity undergoing percutaneous coronary intervention

**DOI:** 10.1186/s12933-019-0952-8

**Published:** 2019-11-09

**Authors:** Annunziata Nusca, Dario Tuccinardi, Claudio Proscia, Rosetta Melfi, Silvia Manfrini, Antonio Nicolucci, Antonio Ceriello, Paolo Pozzilli, Gian Paolo Ussia, Francesco Grigioni, Germano Di Sciascio

**Affiliations:** 1 Unit of Cardiac Sciences, Campus Bio-Medico University of Rome, Rome, Italy; 2 Unit of Endocrinology and Diabetes Department of Medicine, Campus Bio-Medico University of Rome, Rome, Italy; 3 Cardiology Department, Liverpool Heart and Chest Hospital NHS Trust, Liverpool, UK; 4 Cardiovascular and Diabetes Department, IRCCS MultiMedica, Sesto San Giovanni, MI Italy

**Keywords:** Glycated haemoglobin, Glycaemic variability, Continuous glucose monitoring, Platelet reactivity, Percutaneous coronary intervention

## Abstract

**Background:**

Diabetic patients with on-treatment high platelet reactivity (HPR) show an increased risk of thrombotic events. Whether measuring glycated haemoglobin (HbA1c) levels and/or glycaemic variability (GV) may help identifying diabetic patients at higher risk deserving tailored antiplatelet and/or glucose lowering strategies is unknown. We aimed to investigate the relationship between GV, HbA1c levels and platelet reactivity in patients with type 2 diabetes mellitus (DM) undergoing percutaneous coronary intervention (PCI).

**Methods:**

Platelet reactivity was measured in type 2 DM patients using VerifyNow P2Y12 assay. HPR was defined as P2Y12 Reaction Unit (PRU) > 240. GV was expressed through mean amplitude of glycaemic excursions (MAGE) and coefficient of variance (CV) by using the iPro™ continuous glucose recorder.

**Results:**

Thirty-five patients (age 70 ± 9 years, 86% male, mean HbA1c 7.2 ± 1.0%) on clopidogrel therapy were enrolled. HbA1c was independently associated with HPR (OR 7.25, 95% CI 1.55–33.86, p = 0.012). Furthermore, when factored into the model, GV indexes provided independent (OR 1.094, 95% CI 1.007–1.188, p < 0.034) and additional (p < 0.001) diagnostic significance in identifying diabetic patients with HPR.

**Conclusions:**

Glyco-metabolic state significantly correlates with HPR in well-controlled type 2 DM patients on clopidogrel therapy. HbA1c identifies patients at higher thrombotic risk but the highest diagnostic accuracy is achieved by combining GV and HbA1c. Whether individualized antithrombotic and glucose-lowering therapies based on the assessment of these parameters may reduce the incidence of thrombotic events in patients undergoing PCI should be further investigated.

## Background

Patients with type 2 diabetes mellitus (DM) are at higher risk of atherothrombotic events, accountable of about two-thirds of deaths [1, 2]. The enhanced thrombotic status of diabetic patients is related to multiple mechanisms such as endothelial dysfunction, activation of coagulation cascade and high platelet reactivity (HPR) [3]. The latter is considered to play a crucial role in the pathogenesis of macrovascular complications [3], as diabetic patients with HPR showed an over threefold increase in 2-years cardiovascular events recurrence although on steady-state dual antiplatelet therapy [4, 5].

Nevertheless, which glycometabolic parameter should be used in predicting HPR among diabetic patients is unknown. Previous studies investigated the effects of glycated hemoglobin (HbA1c) on platelet function with conflicting results [6–9]. This happens also because fasting blood glucose, fructosamine or HbA1c levels may not offered a complete assessment of glycaemic status [10]. Conversely, glycaemic variability (GV) includes an overall measure of patients’ blood glucose fluctuations, taking into account all hypo- and hyperglycaemic episodes in a pre-determined period of monitoring. Long-term visit-to-visit GV has been shown to be a better risk predictor than mean HbA1c values for micro and macro-vascular complications and all-cause mortality [11]. Moreover, high levels of GV, assessed by continuous glucose monitoring (CGM), appear to have even more deleterious effects than sustained hyperglycaemia in the pathogenesis of cardiovascular autonomic neuropathy and other cardiovascular complications [12, 13]. Previous studies have already correlated an increased GV to the occurrence of adverse events in patients with acute coronary syndromes undergoing percutaneous coronary revascularization (PCI) [14–16]. Finally, critically ill patients admitted to intensive care unit with high GV have shown a significant increased mortality compared with those with less glycemic variability even with slight hyperglycaemia [17].

Notwithstanding, prospective studies investigating the effects of GV on platelet function are lacking. Moreover, whether GV assessment over HbA1c may improve antiplatelet and glucose-lowering management of diabetic patients is still undetermined.

Thus, in this study, we aimed to explore the possible correlation between HbA1c levels and HPR in patients undergoing PCI on clopidogrel therapy. Moreover, we aimed to examine the possible role of GV over HbA1c in identifying diabetic patients at higher thrombotic risk.

## Research design and methods

Consecutive patients with type 2 DM scheduled for elective PCI were prospectively enrolled at our Institution between February and October 2016. Exclusion criteria were: primary intervention for ST-elevation acute myocardial infarction; acute coronary syndrome in the previous 72 h; use of glycoprotein IIb/IIIa inhibitors; platelet count < 70 × 10^9^/l; severe renal failure (glomerular filtration rate < 30 ml/min/1.73 m^2^); coexistent immunological, inflammatory or neoplastic disease at the time of enrolment; high bleeding risk or other contraindications to antiplatelet therapy; availability of glucose sensor for research purpose.

Presence of diabetes mellitus was defined as a history of diabetes controlled by diet or oral glucose-lowering agents or insulin. Coronary intervention was performed with standard technique. All patients were taking aspirin 100–325 mg at the time of the procedure; a 600 mg loading dose of clopidogrel was given at least 2 h before intervention in naïve patients, whereas, patients already on clopidogrel therapy for at least 5 days before the procedure continued to receive a standard dose of 75 mg. After PCI, all patients were maintained on aspirin and clopidogrel 75 mg/day. Our local Ethic Committee approved the study and all subjects enrolled provided written informed consent.

### Platelet function and laboratory testing

**Fig. 1:**
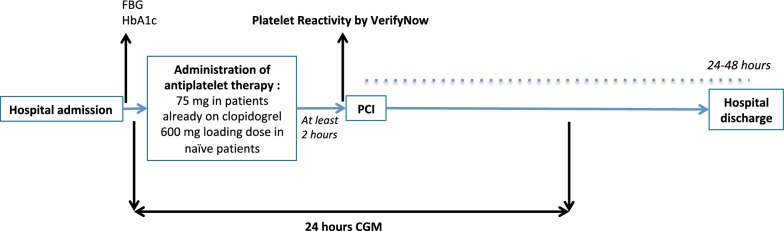
Protocol diagram. *PCI* percutaneous coronary intervention, *FBG* fasting blood glucose, *HbA1c* glycated haemoglobin, *CGM* continuous glucose monitoring

A schematic diagram of the study protocol was reported in Fig. 1. We performed a blood sample in all patients immediately before the percutaneous procedure in the catheterization laboratory, right after the insertion of the radial/femoral sheath, for the assessment of platelet reactivity using the VerifyNow P2Y12 assay (Accumetrics Inc., San Diego, California). Results were expressed as P2Y12 reaction units (PRU) and HPR was defined as a PRU value > 240 [18, 19]. Fasting blood glucose levels and HbA1c were also obtained before PCI in all patients. Furthermore, blood samples were drawn before and at 8 and 24 h after the procedure for creatine kinase-MB (CK-MB) and troponin I (TnI) levels detection; further values were obtained if symptoms suggested myocardial ischemia. These measurements were performed by standard enzymatic procedures (LOCI™ immunochemiluminometric assay—SIEMENS); normal limits were considered 3.6 ng/ml for CK-MB and 0.06 ng/ml for TnI. PCI-related myocardial damage was defined by an elevation of TnI > 5 the 99th percentile URL in patients with normal baseline values and by an increase of TnI > 20% if the baseline values were elevated (> 0.06 ng/ml).

### Glycaemic variability assessment

The glucose monitoring methods were previously reported [20]. Briefly, all enrolled patients were equipped with the iPro™ continuous glucose recorder (Medtronic, Northridge, CA) and a CGM sensor (Enlite^®^ Sensor) was inserted into the subcutaneous abdominal fat tissue about 12 h before the revascularization procedure and platelet reactivity assessment. CGM measured subcutaneous tissue interstitial glucose levels every 5 min, within a range 40–400 mg/dl. The analysis was performed on data obtained in the entire peri-procedural period, from 12 h before to 12 h after PCI. During iPro™ CGM, patients also checked their capillary blood glucose levels with a self-monitoring at least 4 times per day. The FreeStyleLite (Abbott Laboratories, Abbott Park, IL) BG-monitoring system was used to calibrate the iPro™ continuous glucose recorder. After 24-h monitoring, the recorded data were downloaded for the analysis of the glucose excursion parameters using the CareLinkiPro System. Intraday GV was expressed by the GV indexes: standard deviation (SD); coefficient of variation (CV); mean amplitude of glycaemic excursions (MAGE), representing an average size of glycaemic fluctuations; MAGE-up, an average size of glycaemic fluctuations from nadirs to peaks, thus indicating hyperglycaemic excursions; MAGE-down, indicating glycaemic variations from peaks to nadirs, thus hypoglycaemic excursions; continuous overall net glycemic action (CONGA) (1, 2 and 4) assessing magnitude and timing of blood glucose fluctuations captured over various time periods.

### Statistical analysis

Data are presented as percentages and frequencies for categorical variables; whereas, continuous data are presented as mean ± standard deviation (SD). To identify deviations from the normal distribution the Kolmogorov–Smirnov test was used. Comparisons between groups were evaluated using the Mann–Whitney U test, the Chi-square or the Fisher exact test according to distribution. Bivariate correlations between variables were performed and partial correlations were used to adjust for covariates (e.g., BMI and age). HPR and PCI-related myocardial damage were used as dependent variables and only variables which showed a p-value of < 0.1 in the two-way analysis were tested in the hierarchical multivariate model. Results of logistic regression are expressed as odds ratios (ORs) with their 95% confidence intervals (95% CI). Two-tailed p-value < 0.05 was considered statistically significant. The statistical analyses were performed using SPSS version 20.

## Results

Mean age of the overall population was 70 ± 9 years, 33 (94%) of patients underwent elective procedure for stable angina and 17 (49%) were treated by PCI for multivessel coronary disease. Other demographic and clinical characteristics are reported in Additional file 1: Table S1.

High platelet reactivity on clopidogrel therapy was observed in 14 patients (40%). Table 1 reports glycaemic parameters and glucose-lowering therapies of the whole population and according to HPR. At the time of the index procedure the vast majority of patients were treated with metformin and about one-third were on insulin; no difference was observed between the two groups with regard to pre-procedural oral glucose lowering strategies.

**Table 1:** Glycaemic parameters and glucose-lowering therapies in overall population and according to HPR

Variable	Overall population	HPR		p value
		Yes (n = 14)	No (n = 21)	
PRU	207 ± 82	284 ± 35	151 ± 57	< 0.001
Hb1Ac (%)	7.1 ± 1.0	7.9 ± 0.8	6.7 ± 0.8	< 0.001
Hb1Ac (mmol/mol)	54 ± 4	63 ± 2	50 ± 3	
FBG (mg/dl)	131.6 ± 50.8	145.5 ± 38.5	125.4 ± 63.6	0.551
GV indexes				
CONGA 1 (mg/dl)	22.5 ± 8.4	22.7 ± 8.1	22.4 ± 8.9	0.816
CONGA 2 (mg/dl)	31 ± 9.1	30.5 ± 7.5	31.7 ± 11.0	0.856
CONGA 4 (mg/dl)	41.6 ± 13.9	41.9 ± 13.1	41.3 ± 15.4	0.857
MAGE (mg/dl)	66.8 ± 24	80.3 ± 26.9	55.9 ± 14.6	0.009
MAGE-up (mg/dl)	67.7 ± 27.6	71.2 ± 29.9	64.9 ± 26.2	0.938
MAGE-down (mg/dl)	61.4 ± 40.2	52.5 ± 49.4	68.7 ± 30.8	0.421
SD (mg/dl)	34.2 ± 11.4	34.9 ± 11.1	33.6 ± 12.0	0.586
Average glycaemia (mg/dl)	139.0 ± 35.0	140.8 ± 35.9	137.8 ± 36.2	0.897
CV (%)	23.3 ± 5.4	25.9 ± 4.6	21.2 ± 15.3	0.027
Mean duration of diabetes mellitus (months)	41 ± 34	51 ± 9	35 ± 8	0.027
Insulin therapy	12 (36)	7 (21)	5 (15)	0.070
Metformin	20 (60)	8 (23)	12 (35)	0.810
Sulfonylureas	11 (33)	2 (6)	9 (26)	0.090

Values are given as mean ± SD or n (%)

*HPR* high platelet reactivity, *PRU* P2Y12 reaction unit, *Hb1Ac* glycated hemoglobin, *FBG* fasting blood glucose, *GV* glycaemic variability, *CONGA* continuous overall net glycemic action, *MAGE* mean amplitude of glycemic excursions, *SD* standard deviation, *CV* coefficient of variation

**Fig. 2:**
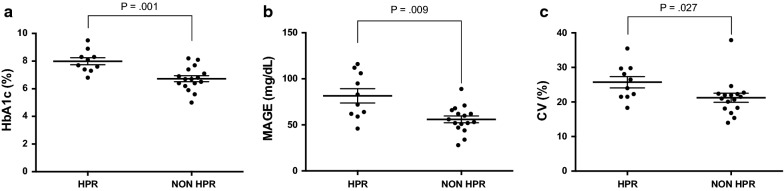
Individual value plots of glycaemic parameters according to on-treatment high platelet reactivity; **a** HbA1c values in patients with and without HPR; **b**, **c** glycaemic variability expressed by MAGE and CV in HPR and non-HPR groups. *HbA1c* glycated haemoglobin, *HPR* high platelet reactivity, *MAGE* mean amplitude glycaemic excursions, *CV* coefficient of variability

Mean HbA1c value was overall consistent with a well-controlled glycaemic status (Table 1). Nevertheless, significantly higher mean HbA1c levels were observed in patients with HPR indicating a worse glycaemic control (p < 0.001; Table 1) (Fig. 2a). Furthermore, increased values of MAGE and CV were also reported in this group (p values ≤ 0.027; Table 1 and Fig. 2b, c).

**Fig. 3:**
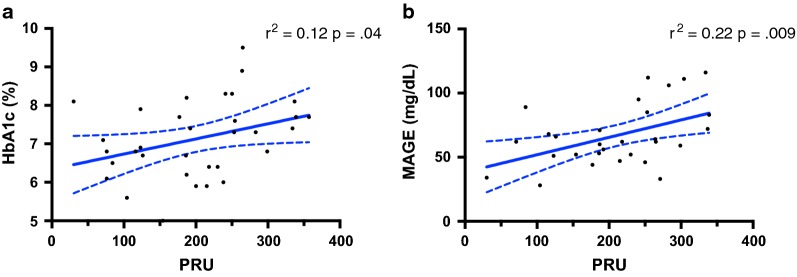
Bivariate correlation plots between glycaemic parameters and PRU; **a** correlation between HbA1c and PRU values (r^2^ = 0.12, p < 0.04); **b** correlation between MAGE and PRU values (r^2^ = 0.22, p < 0.009). *PRU* P2Y12 reaction units, *HbA1c* glycated haemoglobin, *MAGE* mean amplitude glycaemic excursions

At the bivariate analysis, PRU was significantly associated with HbA1c (r^2^ = 0.12, p < 0.04, Fig. 3a) and MAGE (r^2^ = 0.22, p < 0.009, Fig. 3b) levels. Those associations remained significant after controlling for age (HbA1c: Adj r^2^ = 0.12, p < 0.05; MAGE: Adj r^2^ = 0.23, p < 0.01) and BMI (HbA1c: Adj r^2^ = 0.13, p < 0.04; MAGE: Adj r^2^ = 0.19, p < 0.017).

At the multivariate analysis, HbA1c was demonstrated to be an independent predictor of HPR (p < 0.012; Model 1, Table 2). Notably, when MAGE was introduced into the model, it also showed to be an independent marker of HPR even after the adjustment for HbA1c (p < 0.034; Model 2, Table 2). Moreover, introducing MAGE in addition to HbA1c in the hierarchical multivariate model with HPR as the end point, the fit of the model was improved as compared with that including only HbA1c as single predictive variable (pseudoR2: 0.757, p < 0.0001; Model 2, Table 2). Indeed, the combination of MAGE and HbA1c explained the 75% of the variance of HPR compared with the 49% described with HbA1c only.

**Table 2:** Logistic regression models considering HPR as endpoint

	p-value	OR (95% CI)
Model 1 (pseudoR2: 0.499, p < 0.001)		
HbA1c	0.012	7.25 (1.55–33.86)
Model 2 (pseudoR2: 0.757, p < 0.0001)		
HbA1c	0.019	13.21 (1.52–114.19)
MAGE	0.034	1.094 (1.007–1.188)
Model 3 (pseudoR2: 0.618, p < 0.0001)		
HbA1c	0.018	9.13 (1.46–56.79)
CV	0.071	1.22 (0.98–1.53)

*HPR* high platelet reactivity, *Hb1Ac* glycated hemoglobin, *MAGE* mean amplitude glucose excursions, *CV* coefficient of variation

In the multivariate analysis (hierarchical enter method) HPR was entered in the model as dependent variable and as independent variables were included only the variables with p < 0.10 at the bivariate regression analysis: HbA1c, MAGE and CV. Excluded variables: fasting blood glucose, BMI, age, gender, weight, left ventricle ejection fraction, clopidogrel bolus (600 mg), chronic clopidogrel therapy, haemoglobin, haematocrit, platelet count number, white blood cells, total cholesterol, HDL, LDL, triglycerides and all the other glycaemic variability indexes (glycaemic average, SD, CONGA1, CONGA2, CONGA4, MAGE UP, MAGE DOWN); *p < 0.05

Comparable findings were observed when CV was introduced in the hierarchical multivariate model, although without statistical significance (p < 0.071; Model 3, Table 2).

With respect to the effects of GV on clinical outcomes, patients with higher MAGE-up levels showed an increased incidence of PCI-related myocardial damage (p < 0.005, Additional file 1: Table S2), whereas, no difference in HbA1c values were observed between patients with and without post-procedural troponin release. Similarly, MAGE-down tended to increase in patients showing post-procedural myocardial injury, highlighting the unfavourable consequences of hypoglycaemia over hyperglycaemia on myocardial ischemia (p = 0.053, Additional file 1: Table S2). Finally, when MAGE-up was introduced in the hierarchical multivariate model with PCI-induced myocardial damage as the end point, it revealed to be an independent predictor of this complication (p < 0.029, Additional file 1: Table S3).

## Discussion

Our findings indicate a remarkable influence of the glycaemic status on platelet reactivity in type 2 diabetic patients undergoing PCI on clopidogrel therapy. In our series, patients with HPR showed increased HbA1c values as well as higher GV expressed by elevated MAGE and CV levels. Finally, we found an additional prognostic role of GV over HbA1c in identifying diabetic patients at higher thrombotic risk.

### HPR and diabetes

Guidelines recommend that patients with type 2 DM should be treated to achieve a HbA1c level between 7 and 8%, although an even less stringent target has been proposed in those with advanced macrovascular complications, such as established cardiovascular disease [21]. In our population, more than 70% of patients had an HbA1c value ≤ 7%, indicating a well-controlled glycaemic status; however, the incidence of HPR was still 40%. With respect to external validity of the present analysis, these findings are consistent with previous literature reporting reduced responsiveness to antiplatelet drugs in 36–60% of diabetic patients [22–24], with the highest values in those receiving insulin therapy [25]. The high prevalence of HPR among diabetic patients should be properly taken into account on the clinical ground because HPR represents a recognized predictor of post-PCI long-term cardiovascular events, including death, MI and stent thrombosis in patients on antiplatelet therapy [26].

### Glycated haemoglobin and HPR

In our study, HbA1c was observed to be an independent predictor of HPR, with each 1% increase in HbA1c levels translating into a seven-fold rise in the risk of PRU > 240. Previous studies investigating the impact of this glycaemic parameter on platelet reactivity have shown contradictory results [6–9]. Gaborit et al. [6] reported no correlation between ADP-induced platelet aggregation and glycaemic variables such as fasting blood glucose and glycated haemoglobin in a large cohort of diabetic patients. On the other hand, a specifically designed analysis of the prospective, multicenter Assessment of Dual AntiPlatelet Therapy With Drug-Eluting Stent (ADAPT-DES) Registry showed HPR prevalence increasing progressively with HbA1c levels [9]. Unfortunately, no previous study took into account the effects of GV on platelet reactivity.

While HbA1c represents a measure of chronic hyperglycaemia [10, 27], it does not reflect acute glucose fluctuations (hyper/hypoglycaemic spikes). Acute glycaemic variations are closely associated with cardiovascular complications and may be more adequately assessed by using GV [11–17]. Long-term GV has been demonstrated to represent a better glycaemic parameter than mean HbA1c for assessing the risk of micro and macrovascular complications in diabetic patients [11]. Moreover, HbA1c is characterized by several other shortcomings including worse diagnostic performance in different populations such as elderly, patients with iron deficiency anaemia or increased red blood cell turnover, those with end-stage renal disease or alcohol consumption [10].

Consequently, several studies described the clinical impact of GV in patients with coronary artery disease undergoing PCI [14–16]. A high MAGE value has been demonstrated an independent predictor of long-term poor prognosis in a large cohort of patients with acute coronary syndromes undergoing coronary stenting [15].

### Glycaemic variability and HPR

According to our results, GV showed to be relevant in determining platelet reactivity despite an adequate HbA1c level: indeed, at the multivariate analysis, MAGE revealed to be an independent predictor of HPR even after the adjustment for HbA1c. Importantly, GV also exhibited an incremental value over HbA1c in detecting patients at higher thrombotic risk: adding MAGE over HbA1c to the hierarchical multivariate model significantly increases the chance to identify a non-responder to clopidogrel (76% compared with 50% gained with HbA1c measurement only).

To the best of our knowledge, this is the first study investigating the correlation between GV assessed by CGM and platelet reactivity, particularly in the setting of percutaneous coronary revascularization. Previous studies only focused on hyper or hypoglycaemia at one time, failing to demonstrate the biological significance of the combined, which can be only expressed by GV. For instance, induced hypoglycaemia (50 mg/dl) in patients with type 2 DM has been demonstrated to enhance platelet hyperactivity by impairing sensitivity to prostacyclin [28]. On the other side, acute, short-term hyperglycaemia has been proposed to produce transient increased platelet activation after exposure to high shear stress both in vitro and in vivo in diabetic patients, possibly involving enhanced levels of Von Willebrand factor [29]. Furthermore, persistent in vivo platelet activation has been observed in response to postprandial hyperglycaemic spikes in patients with early diagnosed type 2 DM and with HbA1c levels < 7% [30]. Nevertheless, GV is the only measure including positive and negative acute glycaemic fluctuations and, therefore, it might play a more relevant role in promoting a prothrombotic state.

### Glycaemic variability and peri-procedural myocardial damage

In our population higher GV values were observed in patients with PCI-related myocardial damage, confirming our previous evidence that diabetic patients with poor glycaemic control may have a significantly higher risk of suffering from this complication [17]. This is particularly relevant considering PCI-related myocardial injury associated with unfavourable long-term outcome [31]. Interestingly, high levels of MAGE were associated with increased myocardial damage, assessed by myocardial blush grade and ST-segment resolution, also in non-diabetic patients undergoing primary PCI [32].

MAGE-up confirmed to be an independent predictor of this ischemic complication as well as high white blood cells levels. These findings are consistent with experimental studies reporting acute blood glucose fluctuations significantly associated with increased oxidative stress, inflammation and endothelial dysfunction, all factors involved in the pathophysiology of myocardial damage during PCI [33]. Moreover, the occurrence of PCI-associated myocardial damage may reflect a more extensive and unstable atherosclerotic burden, as observed in patients with high GV. An intravascular ultrasound study reported increased MAGE levels in patients with larger plaque volumes and greater lipid plaque components [34].

### Clinical implications

Since HPR is a well-described predictor of adverse thrombotic events [4, 5, 26], our findings underscore the clinical importance of maintaining an adequate glycaemic control, assessed by GV and HbA1c, in diabetic patients undergoing PCI.

For instance, our study generates the hypothesis that periprocedural GV and HbA1c assessment may help to identify those diabetic patients that should be moved towards more aggressive antiplatelet management, such as ticagrelor, also in the setting of an elective procedure [35, 36].

Similarly, given the impact of glycaemic control on HPR, efforts to reduce GV in diabetic patients undergoing PCI may have the same clinical relevance than an intensified platelet inhibition. Indeed, improvement in glucose metabolism assessed by fasting glucose levels, HbA1c and GV has been demonstrated to be associated with reduced oxidative stress, this latter playing an important role in the development of cardiovascular complications in diabetic patients [37]. Moreover, in the last decade, several novel oral glucose lowering drug classes (dipeptidyl peptidase 4 inhibitors, glucagon-like peptide-1 agonists, and sodium-glucose cotransporter 2 inhibitors) have been demonstrated to improve GV indexes [38, 39] and to reduce adverse cardiovascular outcomes in large clinical trials [40]. Few studies have also investigated possible effects of these agents on platelet function with favourable results [41, 42]. Nevertheless, whether the use of these new glucose-lowering drugs may also provide antithrombotic effects improving GV and HbA1c in diabetic patients undergoing PCI should be investigated in further studies.

### Strengths and limitations

The study population was limited, but this is the first study indicating a significant association between GV and HPR. There is not yet a consensus regards the appropriate methods and indexes for assessing GV; consequently our results showing that patients with higher MAGE-up levels had an increased incidence of PCI-related myocardial damage need further confirmation. Nevertheless, in our study we used CV and MAGE for the correlation with HPR, the most accepted metrics for GV according to the previous literature [11, 20]. Furthermore, CGM accuracy may be reduced during the first hours after sensor insertion due to local trauma; however, in our cohort, the mean absolute relative difference (MARD) value, an accepted metric of CGM precision, was < 10% indicating an adequate monitoring performance [43]. CGM was maintained for 24 h, within the 12 h before and the 12 h after PCI; even though a longer period of monitoring might translate in a more precise assessment of GV, nevertheless it would be less applicable to current clinical practice where patients with stable coronary disease are admitted few hours before the procedure and discharged shortly after; we believe that been in keeping with current recommended clinical practice may represent a further strength of the present study. Additionally, GV is likely affected by several pre-, intra- and post-procedural factors in patients undergoing PCI; thus, a 24-h GV monitoring peri-procedural interval may be an adequate time frame taking into account all these aspects and potentially useful, on the clinical ground, for the tailored antiplatelet management of these patients.

Finally, our sample size did not allow to investigate the effects of different glucose lowering agents on GV and, consequently, on platelet reactivity. In addition, we did not explore the effects of glycaemic state on prasugrel or ticagrelor antiplatelet action. However, even if both these two agents are considered the first choice in the acute coronary setting according to guidelines, clopidogrel still remains the more frequently used antiplatelet drug in elective procedures, and we observed as glycaemic state significantly impact patients’ responsiveness to this drug.

## Conclusions

Our hypothesis-generating findings indicate a dangerous but modifiable relationship between glycaemic status assessed by HbA1c levels and GV indexes and HPR in diabetic patients undergoing PCI on clopidogrel therapy. Thus, the present study while underscoring the relevance of a tailored antiplatelet management, on the same time draws our attention to the maintenance of a well-controlled glycometabolic status. Further studies are needed to confirm these results in larger samples.

## Supplementary information

**Additional file 1: Table S1.** Demographic and clinical characteristics of the overall population. **Table S2.** Differences according to PCI-related myocardial damage. **Table S3.** Logistic regression models considering PCI-related myocardial damage as end point.

## Data Availability

The authors declare that all data supporting the findings of this study are available within the article and its Additional file.

## Abbreviations

CGM: continuous glucose monitoring
CK-MB: creatine kinase-MB
CONGA: continuous overall net glycemic action
CV: coefficient of variation
DM: diabetes mellitus
GV: glycaemic variability
HbA1c: glycated hemoglobin
HPR: high platelet reactivity
MAGE: mean amplitude of glycaemic excursions
PCI: percutaneous coronary revascularization
PRU: P2Y12 reaction units
SD: standard deviation
TnI: troponin I
