# Supplementary material for: Incremental role of glycaemic variability over HbA1c in identifying type 2 diabetic patients with high platelet reactivity undergoing percutaneous coronary intervention
Source: Cardiovasc Diabetol. 2019 Nov 9;18:147. doi: 10.1186/s12933-019-0952-8 (PMC6842151; doi:10.1186/s12933-019-0952-8)
Supplement: Supplementary file 1 — Additional file 1: Table S1. Demographic and clinical characteristics of the overall population. Table S2. Differences according to PCI-related myocardial damage. Table S3. Logistic regression models considering PCI-related myocardial damage as end point. [file 12933_2019_952_MOESM1_ESM.docx]

**Additional file 1**

| **Table S1. Demographic and clinical characteristics of the overall population** | |
| --- | --- |
| **N. patients** | 35 |
| Female n. (%) | 5 (14) |
| Age (years) | 70±9 |
| BMI (kg/m^2^) | 28±3 |
| Dyslipidemia n. (%) | 30 (85) |
| Hypertension n. (%) | 33 (94) |
| Smokers n. (%) | 16 (48) |
| Total cholesterol (mg/dl) | 138±35 |
| LDL (mg/dl) | 83±30 |
| HDL (mg/dl) | 41±2 |
| Triglycerides (mg/dl) | 144±14 |
| Creatinine (mg/dl) | 0.9±0.3 |
| eGFR (ml/min/1.73m2) | 88.6±28.4 |
| Stable angina n. (%) | 33 (94) |
| Unstable angina (UA/NSTEMI) n. (%) | 2 (6) |
| Previous MI n. (%) | 15 (43) |
| Multivessel CAD n. (%) | 17 (49) |
| LVEF (%) | 54.0±1.0 |
| Clopidogrel 75 mg n. (%) | 18 (51) |
| Clopidogrel loading dose (600 mg) n. (%) | 17 (49) |

Values are given as mean ± SD or 𝑛 (%). BMI: body mass index; LDL: low density lipoprotein; HDL: high density lipoprotein; eGFR: estimated glomerular filtration rate; UA: unstable angina; NSTEMI: non-ST elevation myocardial infarction; MI: myocardial infarction; CAD: coronary artery disease; LVEF: left ventricular ejection fraction.

| **Table S2 - Differences according to PCI-related myocardial damage** | | | | |
| --- | --- | --- | --- | --- |
| **Variable** | **PCI-MD** | | | **P** |
|  | **Yes (n= 22)** |  | **No (n= 13)** |  |
| Female (%) | 65 |  | 15 | .137 |
| Male (%) | 35 |  | 50 |  |
| Age (years) | 73 ± 11 |  | 67 ± 8 | .182 |
| BMI (kg/m2) | 27 ± 3 |  | 29 ± 3 | .097 |
| Total cholesterol (mg/dL) | 136 ± 44 |  | 135 ± 30 | .737 |
| LDL (mg/dL) | 83 ± 33 |  | 82 ± 26 | .991 |
| HDL (mg/dL) | 36 ± 7 |  | 39 ± 10 | .484 |
| Triglycerides (mg/dL) | 180 ± 135 |  | 140 ± 40 | .979 |
| CRP (mg/dL) | 3.3 ± 3.6 |  | 2.9± 2.3 | .693 |
| HbA1c (%) | 7.42 ± 1.16 |  | 6.95 ± 0.92 | .130 |
| Creatinine (mg/dL) | 0.96 ± 0.39 |  | 0.95 ± 0.29 | .698 |
| eGFR ml/min/1.73m2 | 91 ± 37 |  | 83 ± 26 | .484 |
| Glycaemia (mg/dL) | 114 ± 29 |  | 139 ± 42 | .060 |
| **Glycaemic Variability Indexes** |  |  |  |  |
| CONGA 1 (mg/dL) | 22.3 ± 8.1 |  | 22.1 ± 8.0 | .938 |
| CONGA 2 (mg/dL) | 32.5 ± 9.6 |  | 29.8 ± 7.7 | .551 |
| CONGA 4 (mg/dL) | 43.0 ± 17.0 |  | 40.7 ± 10.7 | .698 |
| MAGE (mg/dL) | 73.4 ± 25.0 |  | 61.0 ± 20.1 | .165 |
| MAGE UP (mg/dL) | 85.2 ± 29.1 |  | 54.3 ± 16.2 | **.005*** |
| MAGE DOWN (mg/dL) | 68.6 ± 64.2 |  | 55.1 ± 15.7 | .053 |
| SD (mg/dL) | 37.1 ± 11.1 |  | 32.3 ± 10.4 | .241 |
| Average Glycaemia (mg/dL) | 137.0 ± 44.0 |  | 138.0 ± 31.0 | .776 |
| CV (%) | 24.5 ± 5.2 |  | 22.0 ± 5.8 | .182 |
| **Clinical presentation** |  |  |  |  |
| Dyslipidemia (%) | 26 |  | 59 | .211 |
| Hypertension (%) | 53 |  | 59 | .282 |
| Smokers (%) | 15 |  | 33 | .550 |
| Stable angina (%) | 32 |  | 62 | .654 |
| Multivessel CAD (%) | 23 |  | 26 |  |
| LVEF (%) | 10.86 ± 8.63 |  | 11.71 ± 7.96 | .737 |
| Hb (g/dl) | 13.6 ± 2.2 |  | 13.3 ± 1.0 | .623 |
| HCT (%) | 40.3 ± 6.1 |  | 39.8 ± 2.9 | .816 |
| Platelet count (10^9^/L) | 205 ± 31 |  | 213 ± 76 | .776 |
| WBC (10^9^/L) | 7.8 ± 2.0 |  | 7.3 ± 1.2 | **.036*** |
| Mean duration of diabetes mellitus (months) | 39 |  | 44 | .643 |
| Insulin treated (%) | 9 |  | 24 | .440 |
| Metformin (%) | 27 |  | 33 | .210 |
| Sulfonylureas (%) | 31 |  | 15 | .280 |

Values are given as mean ± SD or 𝑛 (%). PCI-MD: Percutaneous coronary intervention-myocardial damage; BMI: body mass index; LDL: low density lipoprotein; HDL: high density lipoprotein; CRP: C reactive protein; eGFR: estimated glomerular filtration rate; CONGA: continuous overall net glycaemic action; MAGE: mean amplitude of glycaemic excursions; SD: standard deviation; CV: coefficient of variation; CAD: coronary artery disease; LVEF: left ventricle ejection fraction; Hb: haemoglobin; HCT: hematocrit; WBC: white blood cells.

| **Table S3 - Logistic regression models considering PCI-related myocardial damage as end point** | | | |  |
| --- | --- | --- | --- | --- |
|  |  |  | p-value | OR (95% CI) |
| **Model 1** (pseudoR2: 0.25, p<.021) | | |  |  |
| WBC |  |  | .045 | 1.85 (1.01-3.38) |
|  |  |  |  |  |
| **Model 2** (pseudoR2: 0.68, p<.0001) | | |  |  |
| WBC |  |  | .041 | 3.48 (1.05-11.51) |
| MAGE UP |  |  | .029 | 1.10 (1.01-1.21) |
| WBC: white blood cells; MAGE: mean amplitude of glycaemic excursions. In the multivariate analysis (Hierarchical enter method) PCI-related myocardial damage was entered into the model as dependent variable and as independent variables were included only the variables with p< 0.10 at the bivariate regression analysis: WBC and MAGE-up. Excluded variables: pre-procedure glycaemia, BMI, age, gender, weight, left ventricle ejection fraction, clopidogrel bolus (600 mg), chronic clopidogrel therapy, Hb, HCT, platelet count number, WBC, total cholesterol, HDL, LDL, triglycerides and all the other glycaemic variability indexes (glycaemic average, SD, CONGA1, CONGA2, CONGA4, MAGE UP, MAGE DOWN); *P <.05. | | | | |
|  |  |  |  |  |
|  |  |  |  |  |
